# Increased Expression of Autophagy Protein LC3 in Two Patients With Progressing Chronic Lymphocytic Leukemia

**DOI:** 10.3389/fendo.2020.00321

**Published:** 2020-07-22

**Authors:** Daniela S. Arroyo, Cecilia M. Rodriguez, Claudio Bussi, Clarisa Manzone-Rodriguez, Darío Sastre, Viviana Heller, Carmen Stanganelli, Irma Slavutsky, Pablo Iribarren

**Affiliations:** ^1^Facultad de Ciencias Médicas, Hospital Nacional de Clínicas, Universidad Nacional de Córdoba, Córdoba, Argentina; ^2^Departamento de Bioquímica Clínica, Facultad de Ciencias Químicas, Universidad Nacional de Córdoba, Córdoba, Argentina; ^3^The Francis Crick Institute, London, United Kingdom; ^4^Centro de Investigaciones en Bioquímica Clínica e Inmunología (CIBICI-CONICET), Córdoba, Argentina; ^5^Patología Molecular, Instituto de Investigaciones Hematológicas, Academia Nacional de Medicina, Buenos Aires, Argentina; ^6^Laboratorio de Genética de Neoplasias Linfoides, Instituto de Medicina Experimental, CONICET-Academia Nacional de Medicina, Buenos Aires, Argentina

**Keywords:** chronic lymphocytic leukemia, autophagy, LC3, progressing, cancer

## Abstract

Chronic lymphocytic leukemia (CLL) is the most common type of adult leukemia in the western hemisphere. It is characterized by a clonal proliferation of a population of CD5+ B lymphocytes that accumulate in the secondary lymphoid tissues, bone marrow, and blood. Some CLL patients remain free of symptoms for decades, whereas others rapidly become symptomatic or develop high-risk disease. Studying autophagy, which may modulate key protein expression and cell survival, may be important to the search for novel prognostic factors and molecules. Here, we applied flow cytometry technology to simultaneously detect autophagy protein LC3B with classical phenotypical markers used for the identification of tumoral CLL B cell clones. We found that two patients with progressing CLL showed increased expression of the autophagy protein LC3B, in addition to positive expression of CD38 and ZAP70 and unmutated status of IGHV. Our data suggest that activation of autophagy flux may correlate with CLL progression even before Ibrutinib treatment.

## Introduction

Chronic lymphocytic leukemia (CLL) represents 25% of all leukemias and 1.3% of all cancers in the western hemisphere, but it has lower prevalence in Asia (1, 2). It is characterized by the expansion of a population of monoclonal CD5+ B lymphocytes that accumulate in blood, bone marrow, and secondary lymphoid tissues (3, 4). Chronic lymphocytic leukemia frequently presents with adverse prognostic features in older patients, significantly affecting their survival (1).

The average age at the time of diagnosis is around 70 years old, though, rarely, it is seen in people under age 40. Patients with CLL may be asymptomatic and may initially be diagnosed by the detection of lymphocytosis on a routine complete blood cell count (1). Sometimes the patients develop anemia, thrombocytopenia, lymphadenopathy, and/or hepatosplenomegaly (5). Other cases may present fever, fatigue, night sweats, and weight loss (1). As we describe in a previous report (4), chronic lymphocytic leukemia can present two clinical forms, aggressive and indolent. The worst prognosis is associated with CD38 expression, high expression levels of ZAP-70, and the absence of mutations in the immunoglobulin heavy chain variable (IGHV) genes (4, 6, 7). Discrimination of patients with different outcomes can be done by finding chromosomal alterations, which are normally detected in >80% of cases (4, 8). The most frequent genomic aberration (deletions at chromosome 13q14) can be detected by FISH analysis. Most of the CLL patients show constitutively elevated expression of Bcl-2, indicating a role for resistance to apoptosis in the disease pathogenesis.

The particular ability of autophagy to promote cell survival during metabolic stress or cell death as a result of an imbalance in cell metabolism, where autophagic cellular consumption exceeds the cellular capacity for synthesis, is a promising avenue for cancer therapy (9). As described by Bologna et al. (3), autophagy is activated in leukemia cells upon treatment with different chemotherapeutic agents, inducing cell death. In particular, many currently used drugs for CLL, including fludarabine, dexamethasone, idelalisib, and Bcl-2 antagonists, have been suggested to have an autophagy-mediated effect (3).

It was proposed that conventional CLL prognostic markers like genetic mutations, the mutation status of the IGHV, and expression of ZAP-70 and CD38, have predictive value for the responses to first-line therapy in CLL (4, 10). Many initiatives have aimed at integrating all of the prognostic factors defined previously into a single prognostic score (11, 12). As stated by Strati et al. (1), it has recently been suggested that “published evidence is sufficient to recommend that FISH and IGHV analysis be performed as standard clinical tests for all patients with newly diagnosed CLL in those countries with the resources to do so” (1, 13). Recently, there was a report of differential expression of at least 20 miRs in B cells from progressive CLL patients compared to non-progressive CLL controls (14). Several of these miRs promote resistance to apoptosis and/or progression of neoplastic B cell clones. Therefore, studying cell responses able to modulate miR expression and cell survival, such as autophagy, may be important for the search for novel prognostic factors and molecules (14).

## Background

We present two cases of progressing CLL; the characteristics of the patients are summarized in Table 1. Two additional non-progressing CLL patients are included for comparison purposes. These patients were included in our previous study (4). Here, we describe the patients accordingly to onset, diagnosis, prognostic markers, and evolution.

**Table 1:** Characterization of the CLL patients.

**Sample**	**Age at diagnosis**	**Date of diagnosis**	**Rai stage at diagnosis**	**Gender (male/female)**	**CD38/ZAP70**	**IgVH mutation**	**del 17p**	**del 11q**	**Progression**
CLL#1	58	2008	II	M	+/+	UM	Yes	Yes	Yes
CLL#2	51	2010	IIB	M	+/+	UM	Yes	No	Yes
CLL#3	43	2006	I	F	–/–	MM	No	Yes	No
CLL#4	55	2009	0	M	–/–	MM	Yes	Yes	No

*Chronic lymphocytic leukemia was diagnosed according to standard clinical and laboratory criteria. At the time of analysis, all patients were free from clinically relevant infectious complications. For all in vitro studies, written and informed consent was obtained from patients in accordance with the Declaration of Helsinki. CLL#1 and CLL#2 represent a subgroup of patients with adverse prognostic factors, and CLL#3 and CLL#4 represent patients with more favorable prognostic factors*.

*M, male; F, female; UM, unmutated; MM, mutated*.

### Patient #1

A 58-year-old man was diagnosed with CLL in February 2008. At that time, routine analysis showed a total white blood cell count of 34 × 10^9^/L (normal range: 4.5–10 × 10^9^/L), 82% of lymphocytes (normal range: 20–45%), with splenomegaly and no anemia or thrombocytopenia. Lactate dehydrogenase (LDH) and B2-microglobulin (B2M) values were 312 UI/L and 4.85 mg/L, respectively (reference values: LDH: 180–450 UI/L; B2M: 0.8–2.20 mg/L). Flow cytometry analysis revealed a clonal B cell population with a typical CLL phenotype. Prognostic marker analysis showed 58% of ZAP-70- and 80% of CD38-positive cells (15). Thus, a diagnostic of CLL Rai stage II was done, and the patient was treated with bendamustine from 2009 to 2016 because progressive systemic symptoms were present. At that moment, genetic markers were: 17p deleted 4.7 %, 11q deleted 62.5%, 13q14 14.4%, and the IGHV gene was unmutated. A stringent follow-up was adopted until July 2016, when he arrived at our Center because of progression with lymphadenopathy, hepatosplenomegaly, and systemic symptoms. At this time, he received targeted drug treatment with Ibrutinib for 16 months, achieving partial response. Finally, he progressed to Richter Syndrome, and the patient died 1 month later.

### Patient #2

A 51-year-old man was diagnosed with CLL in February 2010. At that time, routine analysis showed a total white blood cell count of 51.7 × 10^9^/L (normal range: 4.5–10 × 10^9^/L), 91% of lymphocytes (normal range: 20–45%), with hepatosplenomegaly and no anemia or thrombocytopenia. Lactate dehydrogenase (LDH) and B2-microglobulin (B2M) values were 257 UI/L and 3.6 mg/L, respectively (reference values: LDH: 180–450 UI/L; B2M: 0.8–2.20 mg/L). Flow cytometry analysis revealed a clonal B cell population with a typical CLL phenotype, expressing prognostic markers ZAP-70 and CD38 (15). Thus, a diagnostic of CLL Rai stage II was done, and progression was detected in October 2010. In March 2011, first-line treatment of six cycles with Fludarabine, Cyclophosphamide, and Rituximab was administered, followed by two additional cycles in May 2015. Genetic markers were: 17p deleted 20%, 13q 53.9%, 11q normal, and the IGHV gene was unmutated. Relapse was observed in 2014 with profuse sweating and progression to stage IIB. In 2016, further progression to stage IV was observed with weight loss, hepatosplenomegaly, lymphadenopathy, asthenia, sweating, and low platelet count. Ibrutinib treatment was initiated in May 2016, and the patient died in March 2018.

### Patients #3 and #4

Patients 3 and 4 presented non-progressing CLL, RAI stages I and 0, respectively, mutated IGHV gene, and negative for CD38 and ZAP70. They were diagnosed in 2006 and 2009, respectively. They subsequently maintained the typical non-progressive phenotype, and both are still alive (Table 1).

### LC3 Expression in Cells From CLL Patients

**Figure 1:**
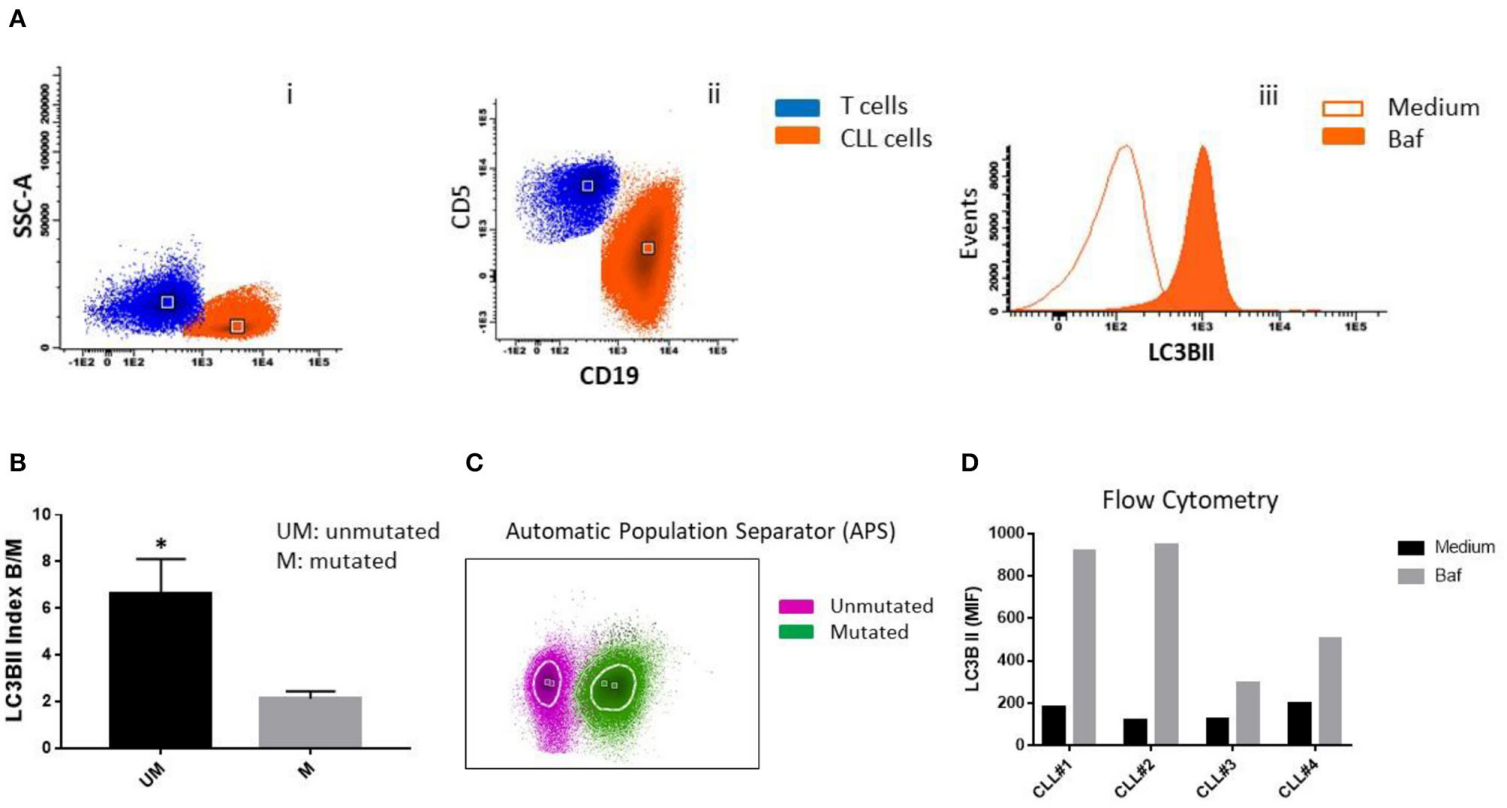
LC3B expression in CLL cells. **(A)** PBMC cells from patients with CLL were treated with 25 nM bafilomycin (Baf) or medium (untreated control) for 24 h. Flow cytometric analysis of LC3B expression in CLL cells is shown. (i) Dot plot of CD19 vs. side scatter (SSC) of B lymphocytes from CLL patients. (ii) Dot plot of CD19 vs. CD5 shows clonal CLL cells and residual T cells. (iii) Histogram showing LC3B expression (MFI) in clonal B cells treated with Baf and untreated cells. **(B)** LC3B index B/M was calculated by dividing MFI of LC3B of CD19+CD5+ (clonal B cells) treated with bafilomycin by MFI of LC3B of untreated clonal B cells (*p < 0.05, unpaired *t*-test). **(C)** Automatic population separator (APS) shows two populations: LC3B+ CLL cells from two patients with unmutated IGHV (Purple) and LC3B+ CLL cells from two patients with mutated IGHV (Green). **(D)** PBMC cells from all four patients (CLL#1, Patient #1; CLL#2, Patient #2; CLL#3, Patient #3; CLL#4, Patient #4) were treated with bafilomycin 25 nM for 2 h. After that, MFI of LC3B was determined by flow cytometry, as described in **(A)**.

At the moments of progression of patients 1 and 2, we evaluated autophagy protein LC3B expression in CLL cells by flow cytometry (FlowCellect^TM^, MilliporeSigma, Darmstadt, Germany). Peripheral blood mononuclear cells (PBMC) from patients with CLL were cultured in the presence or the absence of bafilomycin and then stained using antibodies specific for CD5, CD19, and LC3B. Chronic lymphocytic leukemia B cells were identified and gated using the combination of CD5 plus CD19 staining, for the evaluation of LC3B expression. Lipidated membrane-located LC3B fraction (LC3BII) was accumulated in the presence of bafilomycin (Figure 1A). Samples from non-progressing (mutated IGHV) patients 3 and 4 were also included as controls.

**Figure 2:**
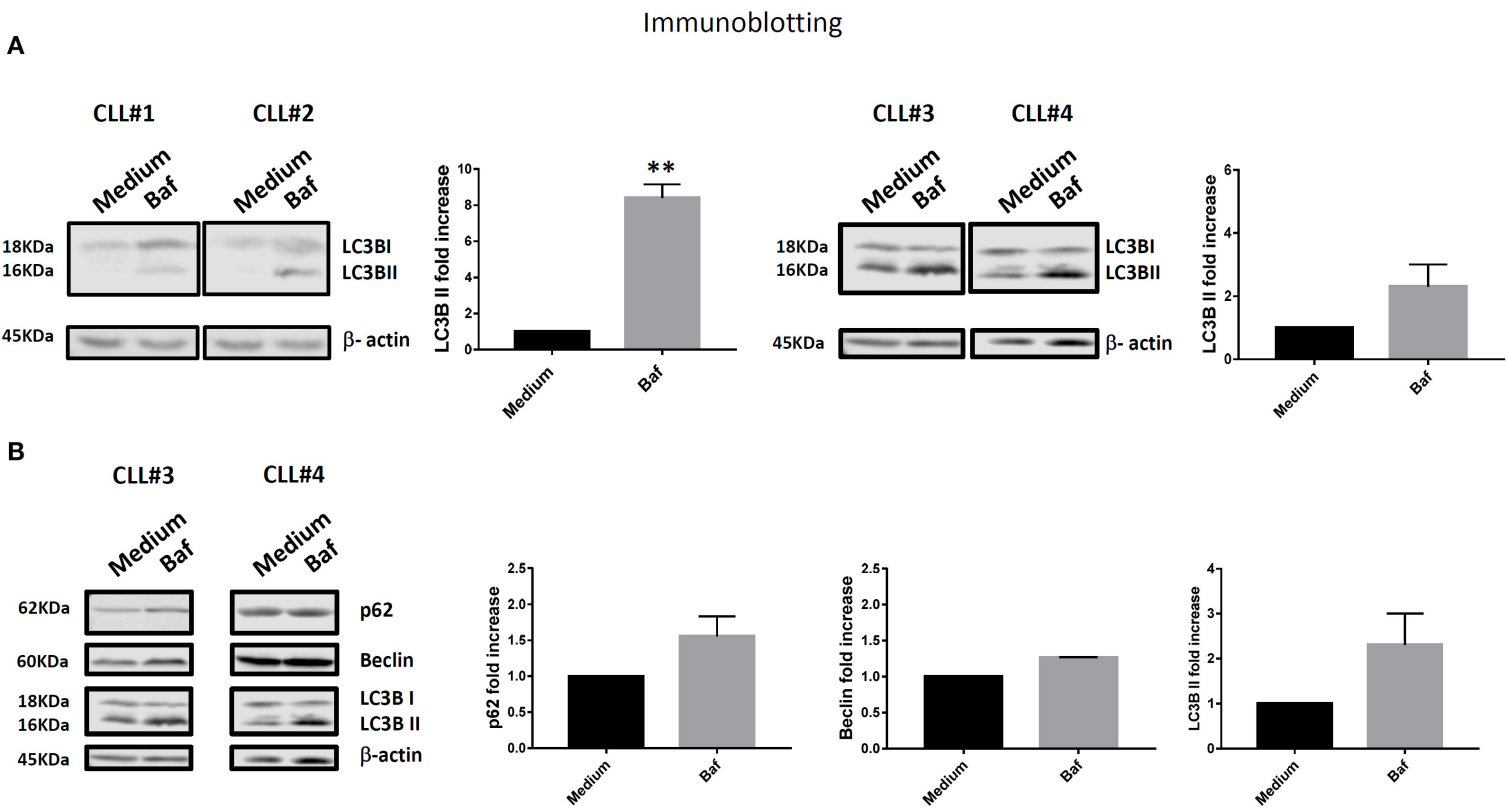
**(A)** PBMC cells from all four patients (CLL#1, Patient #1; CLL#2, Patient #2; CLL#3, Patient #3; CLL#4, Patient #4) were treated as described in Figure 1D, then LC3B and β-actin protein levels were examined by immunoblotting. The figure shows image and relative band intensity quantification. The results were analyzed by *t*-test. Error bars represent SEM (**p < 0.01). **(B)** In addition to LC3B and β-actin, p62 and Beclin protein levels were examined by immunoblotting in new samples from survivor Patients #3 and #4 (CLL#3 and CLL#4, respectively). The results were analyzed by *t*-test. No significant differences were observed.

We observed higher expression of LC3BII in cells from unmutated progressing CLL patients compared to the cells from mutated non-progressing CLL patients (*p* < 0.05) (Figure 1B). In addition, using Infinicyt^TM^ software, we merged files of all the patients into one file and then analyzed immunophenotype-based automatic separation of cell clusters (automatic population separation, APS) (Infinicyt^TM^ software, Cytognos S.L., Salamanca, Spain) based on LC3BII expression (Figure 1C). The APS algorithm clearly discriminated two groups of data corresponding to samples from progressing and non-progressing CLL patients, respectively (Figure 1C). This suggests that LC3BII expression may contribute to the discrimination between progressing and non-progressing patients. In addition, similar results were obtained when LC3BII expression was studied in cells from CLL patients by flow cytometry (Figure 1D) and the classical Western immunoblotting detection of LC3B (Figure 2A). In the presence of bafilomycin, increased LC3BII levels were observed in samples from unmutated progressing CLL patients (Figure 1D). Minimal LC3BII detection was observed in samples from mutated patients (CLL#3 and CLL#4) (Figure 1D). In additional experiments, we analyzed p62/SQSTM1 and Beclin expression in new samples from survivor (mutated IGHV) patients 3 and 4 by Western immunoblotting. We did not find significant differences in the expression levels of autophagy proteins between untreated and bafilomycin-treated samples (Figure 2B).

It is important to notice that increased LC3BII expression was detected by Western immunoblotting (Figure 2A) when the patients progressed but before Ibrutinib treatment. Similar results were obtained by flow cytometry (Figure 1) after Ibrutinib treatment.

These results, taken together, suggest that autophagy activation may correlate with CLL progression beyond Ibrutinib treatment.

## Discussion

Here we found that two patients with progressing CLL showed increased expression of the autophagy protein LC3B in addition to positive CD38 and ZAP70 expression and unmutated status of IGHV.

Autophagy, and autophagy-related proteins (ATG), play a central role in integrating many stress signals to determine the fate of cells (16). There are multiple reports in the literature of autophagy providing resistance to anticancer treatments *in vitro* (17–20), but the resistance mechanisms have yet to be completely determined (16). Autophagy is considered a fundamental survival mechanism that allows cells to adapt to a hostile microenvironment through the recycling of cytosolic molecules in double-membrane vesicles named autophagosomes (21). This mechanism can be induced by several stressors blocking both extrinsic and intrinsic apoptotic pathways (21). It has been described that neoplastic cells can exploit autophagy to survive under hypoxia and low-nutrient conditions (22, 23). Recently, it has become evident that combinatory drug therapy can benefit from the cross-sensitization induced in tumoral cells by cross-modulation of the molecular pathways targeted by each drug. For instance, we recently observed that rapamycin, a mTOR inhibitor, enhanced Fludarabine-induced cytotoxicity in CLL B cells (4). It was reported that pre-treatment of CLL cells with Bruton's tyrosine kinase inhibitor Ibrutinib, whether *ex vivo* or *in vivo* in patients, enhances mitochondrial Bcl-2 dependence, increasing the killing of CLL cells by Venetoclax (24). Similarly, we observed that cells from patients with progressing CLL treated *in vivo* with Ibrutinib were more sensitive to *in vitro* treatment with Venetoclax than cells from patients with non-progressing CLL (data not shown).

Kipps et al. highlighted that the “clinical course of newly diagnosed CLL is very variable; some patients remain free of symptoms and are fully active for decades, whereas others rapidly become symptomatic or develop high-risk disease, which requires treatment soon after diagnosis and might result in death due to therapy-related and/or disease-related complications (2). However, most patients have a clinical course that is in between these two extremes.

More robust prognostic markers are provided by newer techniques, such as flow cytometry, cytogenetics, and molecular biology” (2). Here, we applied flow cytometry technology to simultaneously detect autophagy protein LC3B together with classical phenotypical markers that identify tumoral CLL B cell clones. In addition, we exploited immunophenotype-based (including LC3B expression) automatic separation of cell clusters (APS) to discriminate different groups of data that correlated with the disease progression and IGHV mutational status of the patients. Our results suggest that activation of autophagy flux may correlate with CLL progression even before Ibrutinib treatment. Kong et al. (25) found increased levels of ATG5 and Beclin mRNA in a Chinese cohort of CLL patients compared to healthy controls. However, these results were inconsistent with the findings by Kristensen et al. (26) showing high expression of Beclin being associated with more aggressive disease. The detailed study of the dynamics of autophagosome formation and disappearance during the autophagic flux may solve discrepancies in the interpretation of the role of autophagy in pathogenesis, progression, and therapheutic outcome in CLL.

Autophagy is a very complex response that involves the expression, modification, association, and degradation of ATG proteins. Alterations in the expression of autophagy genes contribute to the tumorigenesis process in numerous types of cancer during tumor initiation, progression, and development and to the maintenance of the malignant state (27). The activation of autophagy flux plays key roles in controlling the tumor microenvironment and in the therapeutic response (28). However, in different subtypes of hematopoietic malignancies, the role of autophagy in cell transformation and the impact of autophagy on the response to different treatment strategies remains ambiguous. In this regard, it has been reported that decreased Bcl-2 levels and an increase in Beclin-1 expression correlate with a favorable clinical outcome in high-grade B-cell lymphomas (29, 30). In line with these observations, it was reported that high expression of LC3 or BECN1 is associated with a favorable outcome in multiple myeloma (31). Nevertheless, other studies have demonstrated an opposite role for the autophagy machinery in disease malignancy development, showing that autophagy activation is required to induce multiple myeloma cell survival (32, 33). Moreover, autophagy machinery activation has been shown to be involved in the progression of other hematopoietic cancers. Indeed, Giatromanolaki et al. reported that patients with follicular and diffuse large B-cell lymphomas express high LC3 levels compared to normal B-cells (34). Considering all of this information, the role of autophagy in lymphoid malignancies is still debated and might be subtype-specific. While several studies have addressed the impact of autophagy in treatment response in several subtypes of hematopoietic malignancies, further studies are needed to better understand the effect of changes in the autophagy flux during disease progression and therapy responses.

## Data Availability Statement

All datasets generated for this study are included in the article/supplementary material.

## Ethics Statement

The studies involving human participants were reviewed and approved by Hospital Nacional de Clínicas, Facultad de Ciencias Médicas, Universidad Nacional de Córdoba, Argentina. The patients/participants provided their written informed consent to participate in this study.

## Author Contributions

DA and CR designed and carried out all the experiments and wrote the manuscript. CB helped with experiments and project discussion. CM-R revised the manuscript. DS helped with sample preparation. VH helped in the organization of the clinical data. CS and IS carried out and helped discuss the results of molecular biology studies. PI conceived and designed the study, supervised all of the experiments, and wrote the manuscript. All authors contributed to the article and approved the submitted version.

## Conflict of Interest

The authors declare that the research was conducted in the absence of any commercial or financial relationships that could be construed as a potential conflict of interest.
